# Thermoelectric properties of graphene-like nanoribbon studied from the perspective of symmetry

**DOI:** 10.1038/s41598-020-66073-y

**Published:** 2020-06-04

**Authors:** Ye-Bin Dai, Kai Luo, Xue-Feng Wang

**Affiliations:** Jiangsu Key Laboratory of Thin Films, School of Physical Science and Technology, Soochow University, 1 Shizi Street, Suzhou, 215006 China

**Keywords:** Physics, Applied physics

## Abstract

We have studied the charge and spin thermopower systematically in a ferromagnetic junction of graphene-like zigzag nanoribbon modified by two on-site disorders in the tight-binding model. Symmetries of the transmission spectra and geometry configuration of the two disorders are important factors in determining the thermoelectric properties of the system. Conditions to achieve pure charge and pure spin thermopower are discussed from the perspective of symmetry. Symmetry breaking is required sometimes to obtain large figure of merit. The type and strength of the disorders can be used to further manipulate the spin polarization of thermal current. Disorders inside nanoribbon instead of on edge can then be used to finely tune the performance of the junction. The results may have great application value in designing thermoelectric devices.

## Introduction

One-dimensional (1D) materials have been demonstrated to be promising systems for high thermoelectric conversion efficiency thanks to the expected reduction of phonon induced thermal conductance^1,2^. Two-probe junctions made of semiconductor nanostructures, molecular wires, or two-dimensional (2D) materials have been proposed to achieve high performance in the last years^3–11^. Specifically, in addition to the traditional charge thermoelectric properties, producing spin current from temperature gradient becomes one of the focuses due to the recent advances in spintronics^12^. Pure thermal spin current with large Seebeck coefficients and figure of merit (*ZT*) is highly pursued^4,6,11–14^.

Graphene has emerged as a prospective 2D material for spintronics because of its long spin relaxation time and length^15^. In addition, special geometry symmetry of graphene can induce spin polarization on edge. Edge magnetism has been confirmed experimentally in zigzag graphene nanoribbons (ZGNRs)^16,17^ and are expected also in graphene-like zigzag nanoribbons (ZNRs). ZNRs can be in ferromagnetic (FM) or antiferromagnetic (AFM) state classified by the relative spin orientations on their two edges^17,18^. External magnetic field can drive a ZNR in its AFM insulator state into its FM metallic state. In this case, charge and spin thermoelectric properties of 1D graphene-like ZNRs have attracted intensive attention in the past decade^4,6,19–28^.

Following the Mott’s formula, pure thermal spin current may be realized at low temperature when the transmission spectra of opposite spins are mirror symmetric with respective to the Fermi level in the energy space. However, the Seebeck coefficients are usually low in perfect intrinsic ZNRs because the slope of transmission spectra vanishes for both spins near the Fermi level. Breaking the geometry symmetry of ZNRs may modulate the energy dependence of transmission^4,19,29,30^ and enhance the Seebeck coefficients^4,5,23,31^. Furthermore, edge disorder may enhance the thermoelectric *ZT* by reducing dramatically phonon thermal transport but affecting only weakly the electronic conduction^23^. Chemical and physical modifications have been proposed to obtain demanded thermoelectric properties in this principle. It was predicted that combination of *n*- and *p*-type doping on opposite edges of ZGNRs can boost the spin thermoelectric effect^4^. Edge defects can also lead to the occurrence of spin-dependent Seebeck effect and the enhancement of charge and spin *ZT*^5^. A strong reduction of thermal conductance compared with the single graphene nanoribbon has been predicted in twisted bilayer graphene nanoribbon junctions and outstanding *ZT* values may be achieved in some specific configurations^31^.

Disorders can also be introduced manually by applying external electric potential in the range of atomistic scale employing state-of-art techniques, such as the scanning tunneling microscopy (STM) and atomic force microscopy^32,33^. This allows continuous variation of disorder parameters and facilitates systematic investigation on the effects of disorder configuration and profile. In a previous work, using a tight-binding model for FM ZNRs, we have studied effects of local potential at a single site on the charge and spin thermopower and obtained inspiring result^20^. Comparison with the first-principles calculation of edge doped ZGNRs shows that boron atom doping corresponds to add an external on-site potential of 3.24 eV.

In this work, we will discuss effects of two on-site disorders on the thermoelectric properties of FM ZNRs. The symmetry of the disorder configuration is found a key to achieve high thermoelectric performance. Properly choosing disorder profile and obtaining transmission spectra with desired symmetry, we can design systems for pure charge or pure spin thermopower with high Seebeck coefficient and high *ZT* value.

## Models and Methods

**Figure 1:**
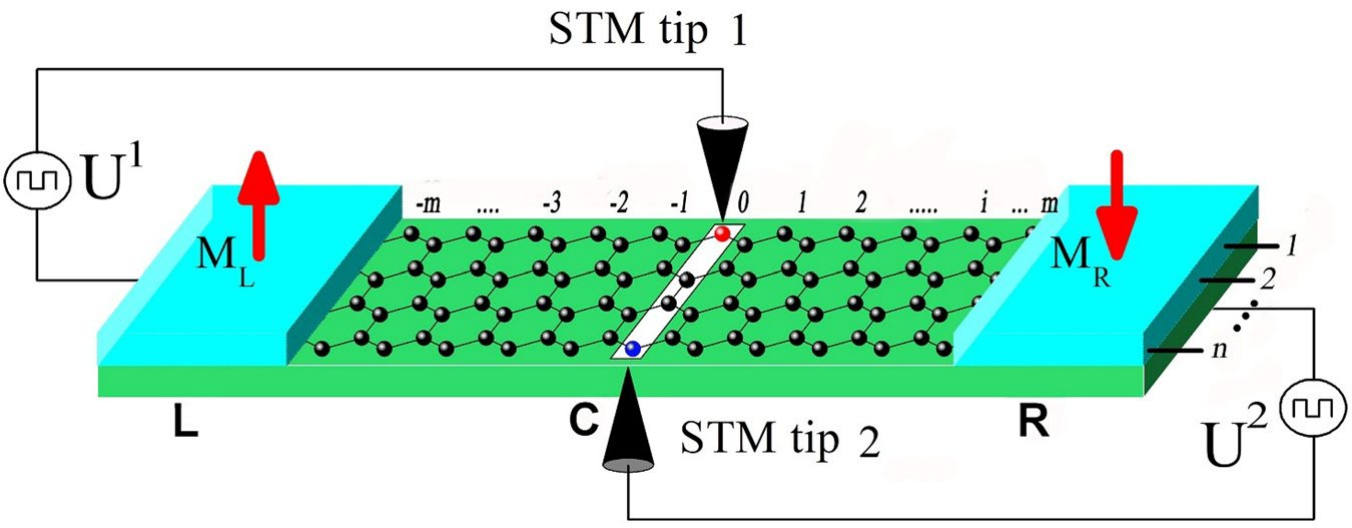
Schematic structure of a FM *n*-ZNR two-probe junction with a central region of length 2 *m*. Two local potentials ${U}_{{i}_{\alpha },{j}_{\alpha }}^{\alpha }$ for $\alpha =1,2$ are applied on separate sites $({i}_{\alpha },{j}_{\alpha })$ via two STM tips. ${i}_{\alpha }\in [-m,m]$ and ${j}_{\alpha }\in [1,n]$ indicate the longitudinal and lateral coordinates of the cites.

We consider a FM ZNR in the tight-binding model with external local potentials applied on two separate sites via STM tips. As can be seen from Fig. 1, a two-probe junction is established by partitioning the ZNR into the left electrode (L), the central device region (C), and the right electrode (R). The two electrodes can be magnetized with parallel (*p*) and antiparallel (*ap*) magnetizations to make the system into *p* and *ap* junctions, respectively. The Hamiltonian reads^34^:

1 $$H=\sum _{h,\sigma }\sigma {M}_{h}{c}_{h,\sigma }^{\dagger }{c}_{h,\sigma }-\sum _{h,l,\sigma }t({c}_{h,\sigma }^{\dagger }{c}_{l,\sigma }+h.c.\,)+\sum _{\alpha ,\sigma }{U}_{{k}_{\alpha }}^{\alpha }{c}_{{k}_{\alpha },\sigma }^{\dagger }{c}_{{k}_{\alpha },\sigma }$$

where ${c}_{h,\sigma }^{\dagger }({c}_{h,\sigma })$ are the creation (annihilation) operators for electrons on site $h$, with spin index $\sigma =-\,1(\,\uparrow \,)$ or $+1(\,\downarrow \,)$. The uniform on-site energy of the corresponding pristine ZNRs is set to zero. $T$ is the nearest-neighbor hopping integral and is chosen as the energy unit in this paper with $t=2.7{\rm{eV}}$ for ZGNRs. ${U}_{{k}_{\alpha }}^{\alpha }$, with ${k}_{\alpha }\equiv ({i}_{\alpha },{j}_{\alpha })$ and $\alpha =1,2$ for the convenience, is the $\alpha -{\rm{th}}$ extrinsic local potential energy on site ${k}_{\alpha }$ which may originate from impurities, defects, STM tips, or other disorder sources. ${i}_{\alpha }\in [-m,m]$ and ${j}_{\alpha }\in [1,n]$ denote the longitudinal and lateral coordinates, respectively, in the central region. $\sigma {M}_{h}$ remarks the on-site Zeeman energy due to the edge magnetization and has the same value on the two edges. It decays linearly in the lateral (*y*) direction from the value on the edges to zero in the mid line of the ribbon. Along the longitudinal (*x*) direction on edges, $\sigma {M}_{h}$ has a uniform maximal value $\sigma M$ (full magnetization) in the electrodes. In region C, $\sigma {M}_{h}=\sigma M$ on edges in *p* junctions but changes linearly from $\sigma M$ to $-\sigma M$ in *ap* junctions.

The electron spin-dependent current ${I}_{\sigma }$ in the Landauer-Buttiker formalism without non-coherent effects is given by:

2 $${I}_{\sigma }=\frac{e}{h}\int d\varepsilon [{f}_{L\sigma }(\varepsilon )-{f}_{R\sigma }(\varepsilon )]{\tau }_{\sigma }(\varepsilon )$$

where ${f}_{\beta \sigma }(\varepsilon )=1/\{\exp [(\varepsilon -{\mu }_{\beta \sigma })/{k}_{B}{T}_{\beta }]+1\}$ is the Fermi-Dirac distribution of electrons in the $\beta$ electrode and ${T}_{\beta }$ the temperature^35^. In this paper we focus on thermally related effects in the linear response regime near Fermi level ${\mu }_{\beta \sigma }=0$ and neglect the effect of local potentials on ${\mu }_{\beta \sigma }$. The transmission is given by ${\tau }_{\sigma }(\varepsilon )=Tr{[{\varGamma }_{L}{G}^{r}{\varGamma }_{R}{G}^{a}]}_{\sigma }$. ${G}^{r}({G}^{a})$ is the retarded (advanced) Green function corresponding to the Hamiltonian in region C: ${G}^{r}(\varepsilon )={[{G}^{a}(\varepsilon )]}^{+}={[\varepsilon -{h}_{C}-{\varSigma }_{L}-{\varSigma }_{R}]}^{-1}$, and ${\varGamma }_{L(R)}(\varepsilon )=i[{\varSigma }_{L(R)}-{\varSigma }_{L(R)}^{+}]$ is the broadening function. Besides, the iterative procedure gives the self-energy function ${\varSigma }_{L(R)}$ which is the result of coupling between the device and electrode.

In the linear response regime of a small spin-dependent voltage bias $\Delta {V}_{\sigma }$ and a small temperature difference $\Delta T$ between the electrodes, we expand $\varDelta f={f}_{L}-{f}_{R}$ in a Taylor series and obtain ${I}_{\sigma }={G}_{0}{K}_{0\sigma }({\mu }_{\sigma },\,T)\Delta {V}_{\sigma }\,+\,{G}_{0}{K}_{1\sigma }({\mu }_{\sigma },\,T)\Delta T/(eT)$ with the conductance unit ${G}_{0}={e}^{2}/h$ and ${K}_{v\sigma }({\mu }_{\sigma },T)=-\,\int d\varepsilon \,[\partial f(\varepsilon ,{\mu }_{\sigma },T)/\partial \varepsilon ]\,{(\varepsilon -{\mu }_{\sigma })}^{v}{\tau }_{\sigma }(\varepsilon )$, for $v=0,1,2$. The corresponding charge (${S}_{c}$) and spin (${S}_{s}$) Seebeck coefficients, for an open circuit ${I}_{\sigma }=0$, are given by ${S}_{c}=({S}_{\uparrow }+{S}_{\downarrow })/2$ and ${S}_{s}={S}_{\uparrow }-{S}_{\downarrow }$, respectively, with

3 $${S}_{\sigma }=-\,\mathop{\mathrm{lim}}\limits_{\Delta T\to 0}\frac{\Delta {V}_{\sigma }}{\Delta T}=-\frac{1}{eT}\frac{{K}_{1\sigma }({\mu }_{\sigma },T)}{{K}_{0\sigma }({\mu }_{\sigma },T)}.$$

The Mott’s formula ${S}_{\sigma }\approx -({\pi }^{2}{k}_{B}^{2}T/3e)\,{\tau {\prime} }_{\sigma }({\mu }_{\sigma })/{\tau }_{\sigma }({\mu }_{\sigma })$ can be used to obtain analytical results at low temperatures^36–39^. In general, we calculate the electron conductance ${G}_{c}={G}_{\uparrow }+{G}_{\downarrow }$, the spin conductance ${G}_{s}={G}_{\uparrow }-{G}_{\downarrow }$, and the electron thermal conductance ${\kappa }_{e}={\kappa }_{e\uparrow }+{\kappa }_{e\downarrow }$ from ${G}_{\sigma }={G}_{0}{K}_{0\sigma }({\mu }_{\sigma },T)$ and ${\kappa }_{e\sigma }=\frac{1}{hT}\left\{{K}_{2\sigma }({\mu }_{\sigma },T)-\frac{{K}_{1\sigma }{({\mu }_{\sigma },T)}^{2}}{{K}_{0\sigma }({\mu }_{\sigma },T)}\right\}$^40^. The charge and spin thermoelectric figures of merit can then be defined as^41–43^

4 $${Z}_{c}T=\frac{{G}_{c}{S}_{c}^{2}T}{{\kappa }_{e}+{\kappa }_{ph}}\,{\rm{and}}\,{Z}_{s}T=\frac{|{G}_{s}|{S}_{s}^{2}T}{{\kappa }_{e}+{\kappa }_{ph}}$$

where ${\kappa }_{ph}$ denotes the thermal conductance due to the phonon contribution. The spin polarization of current is characterized by $SP=({I}_{\uparrow }-{I}_{\downarrow })/({I}_{\uparrow }+{I}_{\downarrow })$^44^.

The model should be valid if it can mimic the band structure around the Fermi level of a nanoribbon system. In a pristine ZNR *t* determines the band shape and *M* gives the band separation of opposite spins at the Brillouin zone edge. In systems with disorders such as dopants, defects, and applied gate voltages, the profile of on-site energies ${U}_{{k}_{\alpha }}^{\alpha }$ describes their effects. It has been shown that an edge dopant of single boron atom in ZGNRs gives a *U* around 3 eV^20^.

## Result and Discussion

The symmetry of transmission spectrum play important role in determining the thermoelectric properties of *n*-ZNR two-probe junctions with even *n*. The three mainly concerned symmetries are ① Spin degeneracy, i.e. ${\tau }_{\sigma }(\varepsilon )={\tau }_{\bar{\sigma }}(\varepsilon )$; ② Mutual mirror of opposite spins with respect to the Fermi level, i.e. ${\tau }_{\sigma }(\varepsilon )={\tau }_{\bar{\sigma }}(\,-\varepsilon )$ with $\bar{\sigma }=-\,\sigma$; and ③ Mirror symmetry, i.e. ${\tau }_{\sigma }(\varepsilon )={\tau }_{\sigma }(\,-\varepsilon )$ for both spins. In the following we will use a symmetry set (①,②,③) to describe the symmetry properties of any spectrum with symmetry value 1 for the presence and 0 for usual absence of the symmetry.

The symmetry of transmission spectra can determine the thermoelectric properties qualitatively according Eqs. (2) and (3). At first, the spin thermoelectric effect vanishes with ${S}_{s}=0$ in the presence of symmetry ①. Secondly, symmetry ②leads to the disappearance of the charge thermoelectric effects. Since $\partial f/\partial \varepsilon$ is an even function of $\varepsilon$ and ${\tau }_{\uparrow }(\varepsilon )={\tau }_{\downarrow }(\,-\,\varepsilon )$, we have ${K}_{\nu \uparrow }({\mu }_{\sigma },T)={(-1)}^{\nu }{K}_{\nu \downarrow }({\mu }_{\sigma },T)$, ${S}_{\uparrow }=-\,{S}_{\downarrow }$, and ${S}_{c}=0$. Finally, both charge and spin thermoelectric effects vanish in the presence of symmetry ③. In this case ${\tau }_{\sigma }(\varepsilon )$ is an even function while $[{f}_{L\sigma }(\varepsilon )-{f}_{R\sigma }(\varepsilon )]$ is an odd function in the existence of temperature gradient. So the thermal current is zero following Eq. (2).

### Symmetry of transmission spectra

The symmetry of transmission spectra is closely relevant to the geometry configuration of disorders. We consider at first edge disorders when two local potentials of constant magnitude $|{U}_{{i}_{1},{j}_{1}}^{1}|=|{U}_{{i}_{2},{j}_{2}}^{2}|=t$ are applied on edge with ${j}_{1},{j}_{2}=$ 1 or *n*. The disorder configurations of ${U}_{{i}_{1},{j}_{1}}^{1}$ and ${U}_{{i}_{2},{j}_{2}}^{2}$ are classified according to their 1) **lateral positions**: on the same edge with ${j}_{1}{j}_{2}=1,{n}^{2}$ or on opposite edges with ${j}_{1}{j}_{2}=n$; 2) **longitudinal positions**: on the same side with ${i}_{1}{i}_{2} > 0$, on opposite sides with ${i}_{1}{i}_{2} < 0$, one at the midpoint with ${i}_{1}=0,\,{i}_{2}\ne 0$, or both at the midpoint with ${i}_{1}={i}_{2}=0$; and 3) **signs**: the same sign with ${U}_{{i}_{1}{j}_{1}}^{1}{U}_{{i}_{2}{j}_{2}}^{2} > 0$ or opposite signs with ${U}_{{i}_{1}{j}_{1}}^{1}{U}_{{i}_{2}{j}_{2}}^{2} < 0$.

Under *p* junction, the transmission spectra are usually spin nondegenerate and has no mirror symmetry. We observe symmetry set (0,1,0) in the transmission spectra of *n*-ZNRs when applying a positive local potential and a negative one with ${U}_{{i}_{1}{j}_{1}}^{1}{U}_{{i}_{2}{j}_{2}}^{2} < 0$. Symmetry set (0,0,0) appears for ${U}_{{i}_{1}{j}_{1}}^{1}{U}_{{i}_{2}{j}_{2}}^{2} > 0$ except for 2-ZNR in cases ${i}_{1}{i}_{2} > 0$ and ${i}_{1}={i}_{2}=0$ with ${j}_{1}{j}_{2}=n$ where (0,1,0) is observed instead.

Under *ap* junction, the edge magnetization changes sign in region C and offers more symmetry choices of transmission spectrum for thermoelectric manipulation. In Table 1 we list the interesting symmetries observed in transmission spectra for different disorder configurations in case $|{i}_{1}|=|{i}_{2}|$. The result in case $|{i}_{1}|\ne |{i}_{2}|$ is not shown since no symmetry can be observed usually and we have trivially symmetry set (0,0,0). We will assume $|{i}_{1}|=|{i}_{2}|$ in the rest of the paper if not specified.

**Table 1:** Transmission spectrum symmetries (①,②,③) of an *n*-ZNR *ap* junction for even *n* but different disorder configurations. Configurations of the two edge-site disorders ($\alpha =1,2$) are described by their lateral positions ${j}_{\alpha }$ (1 for upper edge and *n* for lower edge), longitudinal positions ${i}_{\alpha }$ with $|{i}_{1}|=|{i}_{2}|$ (negative for left side and positive for right side), and sign of ${U}_{{i}_{\alpha },{j}_{\alpha }}^{\alpha }$ with $|{U}_{{i}_{1},{j}_{1}}^{1}|=|{U}_{{i}_{2},{j}_{2}}^{2}|$.

${j}_{1}{j}_{2}=$	${i}_{1}{i}_{2}$	${U}_{{i}_{1}{j}_{1}}^{1}{U}_{{i}_{2}{j}_{2}}^{2}$	①	②	③
$1\,{\rm{or}}\,{n}^{2}$	<0	>0	1	0	0
		<0	0	0	1
*n*	>0	>0	0	0	0
		<0	0	1	0
	<0	>0	1	0	0
		<0	0	0	1
	${i}_{1}={i}_{2}=0$	>0	1	0	0
		<0	1	1	1

The presence and absence of symmetry is denoted by 1 and 0, respectively.

**Figure 2:**
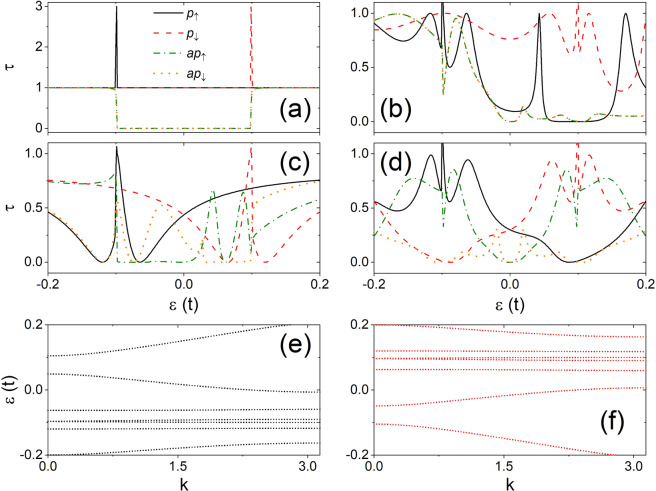
Transmission spectra of spins $\uparrow$ and $\downarrow$ under *p* (solid and dashed) and *ap* (dash-dotted and dotted) junction in a two-probe junction of 4-ZNR with $m=5,M=0.1t$ for (**a**) ${U}^{1}={U}^{2}=0$, (**b**) ${U}_{5,1}^{1}=t,{U}_{-5,1}^{2}=t$, (c) ${U}_{4,1}^{1}=t,{U}_{5,1}^{2}=-\,t$, and (**d**) ${U}_{5,1}^{1}=t,{U}_{-5,1}^{2}=-\,t$. The energy bands of a virtual bulk ZNR with supercell the same as region C of the configuration in (**c**) under *p* junction are also plotted in (**e**) for spin spins $\uparrow$ and in (**f**) for spin $\downarrow$.

As an example, we present the transmission spectra of a 4-ZNR junction with $m=5$ and $M=0.1t$ in Fig. 2 under junctions *p* and *ap* for four typical disorder configurations. In the absence of local potential the transmission spectra are fully determined by the energy band and they have the same symmetry as shown in Fig. 2(a). The spin up (down) transmission spectrum in case *p* is a $\tau =1$ platform except the sharp peak at $\varepsilon =-\,M(M)$ and has the symmetry (0,1,0). This peak originates from the twist of energy band in agreement with the results obtained from the DFT simulation for zigzag nanoribbons of graphene^4,45^, silicone^46^, and zigzag $\alpha -$ graphyne^19^. In case *ap*, however, the lateral wave function of $\pi$ state in one electrode is orthogonal to that of ${\pi }^{\ast }$ state in the other electrode for the same spin orientation and the electrons near the Fermi level cannot tunnel between the two electrodes^4,19,45,46^. As a result, the transmission spectrum become $\tau =1$ platforms with a $\tau =0$ gap in range of $\varepsilon \in [\,-\,M,M]$ and has symmetry (1,1,1).

In the existence of disorders, transmission dips may appear due to the Fano effect arising from the formation of impurity bound states. Theses characteristic transmission dips have been confirmed by DFT simulations in FM nanoribbons of graphene-like-materials^4,19,20,45,46^. In addition, the disorders in the central region can break the geometric symmetry of the system and couple the orthogonal wave functions between the electrodes in case *ap*. This narrows the conductance gap near the fermi level as also confirmed by DFT simulation^19,20,46^. In Fig. 2(b), we plot the transmission spectra in junction of two positive local potentials with ${U}_{{i}_{1}{j}_{1}}^{1}{U}_{{i}_{2}{j}_{2}}^{2} > 0$ in the case ${j}_{1}{j}_{2}=1$, and ${i}_{1}{i}_{2} < 0$. Under *p* junction, no symmetry appears in the spectra. Under *ap* junction, the spectra have symmetry set (1,0,0) with symmetry ① satisfied. Similarly, symmetry ① also exists in the spectra for ${U}_{{i}_{1}{j}_{1}}^{1}{U}_{{i}_{2}{j}_{2}}^{2} > 0$ in case ${j}_{1}{j}_{2}={n}^{2}$ and ${i}_{1}{i}_{2} < 0$, ${j}_{1}{j}_{2}=n$ and ${i}_{1}{i}_{2} < 0$, or ${j}_{1}{j}_{2}=n$ and ${i}_{1}={i}_{2}=0$, and for ${U}_{{i}_{1}{j}_{1}}^{1}{U}_{{i}_{2}{j}_{2}}^{2} < 0$ the case ${j}_{1}{j}_{2}=n$ and ${i}_{1}={i}_{2}=0$ as shown in Table 1.

The transmission spectra in the presence of a positive and another negative local potential with ${U}_{{i}_{1}{j}_{1}}^{1}{U}_{{i}_{2}{j}_{2}}^{2} < 0$, ${j}_{1}{j}_{2}=1$, and ${i}_{1}{i}_{2} > 0$ are illustrated in Fig. 2(c). Under *p* junction, the transmission spectra are determined by the energy spectra of electrons in region C. We establish a virtual bulk ZNR with unit cell the same as region C of the configuration used in Fig. 2(c) and calculate its energy band for reference as illustrated in Fig. 2(e) and (f). The mutual mirror symmetry of opposite energy bands with respect to the Fermi level is not broken and the transmission spectra show symmetry set (0,1,0) with symmetry ② satisfied. Under *ap* junction, symmetry ② is usually broken and symmetry set (0,0,0) is obtained in the case of Fig. 2(c). However, symmetry ② of the spectra may appear in *ap* junction in case ${U}_{{i}_{1}{j}_{1}}^{1}{U}_{{i}_{2}{j}_{2}}^{2} < 0$, ${j}_{1}{j}_{2}=n$, and ${i}_{1}{i}_{2}\ge 0$ as shown in Table 1. Symmetry ② also appears in the spectra in *p* junction if ${U}_{{i}_{1}{j}_{1}}^{1}{U}_{{i}_{2}{j}_{2}}^{2} < 0$.

The transmission spectra in case ${U}_{{i}_{1}{j}_{1}}^{1}{U}_{{i}_{2}{j}_{2}}^{2} < 0$, ${j}_{1}{j}_{2}=1$, and ${i}_{1}{i}_{2} < 0$ are illustrated in Fig. 2(d). Under *p* junction, the transmission spectra show symmetry set (0,1,0) for the same reason discussed in Fig. 2(c). Under *ap* junction, the spectra is described by symmetry set (0,0,1) with symmetry ③ satisfied. As illustrated in Table 1, symmetry ③ also appears on the spectra if ${U}_{{i}_{1}{j}_{1}}^{1}{U}_{{i}_{2}{j}_{2}}^{2} < 0$ in case ${j}_{1}{j}_{2}={n}^{2}$ and ${i}_{1}{i}_{2} < 0$, ${j}_{1}{j}_{2}=n$ and ${i}_{1}{i}_{2} < 0$, or ${j}_{1}{j}_{2}=n$ and ${i}_{1}={i}_{2}=0$.

### Pure charge or pure spin thermopower

To achieve pure charge thermal current, we look for systems having transmission spectra with symmetry ① and without symmetry ② and ③, i.e. symmetry set (1,0,0). As illustrated in Table 1, symmetry ① can be satisfied only in four cases under the *ap* junction. However, the current may vanish in case ${j}_{1}{j}_{2}=n$, ${i}_{1}={i}_{2}=0$, and ${U}_{{i}_{1},{j}_{1}}^{1}{U}_{{i}_{2},{j}_{2}}^{2} > 0$ due to the wide transmission gap at the Fermi level, and in case ${j}_{1}{j}_{2}=n$, ${i}_{1}={i}_{2}=0$, and ${U}_{{i}_{1},{j}_{1}}^{1}{U}_{{i}_{2},{j}_{2}}^{2} < 0$ since ${S}_{c}$ and ${S}_{s}$ become zero for transmission spectra with symmetries ①, ② and ③. Therefore, pure thermal charge current with ${S}_{c}\ne 0$ and ${S}_{s}=0$ can be observed in the two cases when ${i}_{1}{i}_{2} < 0$ and ${U}_{{i}_{1},{j}_{1}}^{1}{U}_{{i}_{2},{j}_{2}}^{2} > 0$.

**Figure 3:**
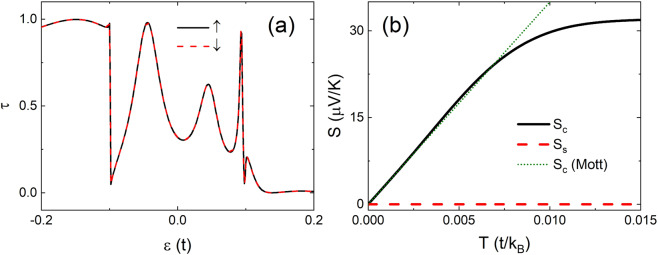
(a) Transmission spectra and (**b**) Seebeck coefficients versus the temperature of a 4-ZNR a*p* junction with $m=5,M=0.1t$, and ${U}_{3,1}^{1}={U}_{-3,4}^{2}=t$ are presented. ${S}_{c}$ estimated from the Mott’s formula is show by the dotted line.

In Fig. 3(a,b) we present transmission spectra and Seebeck coefficients versus temperature, respectively, in a 4-ZNR junction with $m=5$ and $M=0.1t$ for ${U}_{3,1}^{1}={U}_{-3,4}^{2}=t$ under *ap* junction. A large transmission peak appears at $\varepsilon =-\,0.044t$ while a small one appears at $\varepsilon =0.045t$. The transmission spectra satisfy symmetry ① but not ② and ③. ${S}_{c}$ shows linear dependence on $T$ (solid) with $d{S}_{c}/dT\approx 3498\mu {k}_{B}{\rm{V}}/t{\rm{K}}$ in agreement with the Mott’s formula (dotted) at low temperature and then saturates at high temperature $T > 0.075t/{k}_{B}$. This happens when the nonlinear spectra play a great role at the high temperature. Interestingly ${S}_{s}$ is strictly zero in the whole range of temperature, and the pure charge thermal current is protected by the symmetry of the system.

Pure spin thermal current appears for transmission spectra with symmetry ② but without ① and ③, i.e. symmetry set (0,1,0). Symmetry ② can be satisfied only in three cases as shown in Table 1. However, the current may vanish in case ${j}_{1}{j}_{2}=n$, ${i}_{1}={i}_{2}=0$, ${U}_{{i}_{1}{j}_{1}}^{1}{U}_{{i}_{2}{j}_{2}}^{2} < 0$ under the *ap* junction since ${S}_{c}$ and ${S}_{s}$ become zero for transmission spectra with symmetries set (1,1,1). Therefore, pure thermal spin current with ${S}_{s}\ne 0$ and ${S}_{c}=0$ can be observed only in cases ${U}_{{i}_{1}{j}_{1}}^{1}{U}_{{i}_{2}{j}_{2}}^{2} < 0$ under the *p* junction and ${j}_{1}{j}_{2}=n$, ${i}_{1}{i}_{2} > 0$, ${U}_{{i}_{1}{j}_{1}}^{1}{U}_{{i}_{2}{j}_{2}}^{2} < 0$ under the *ap* junction.

**Figure 4:**
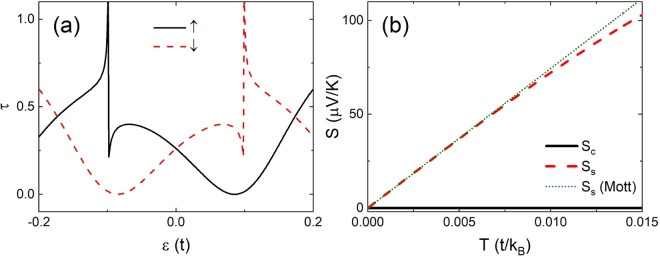
(**a**) Transmission spectra and (**b**) Seebeck coefficients versus temperature in a 4-ZNR *p* junction with $m=5$, $M=0.1t$, ${U}_{0,1}^{1}=t$, and ${U}_{0,4}^{2}=-\,t$. ${S}_{s}$ from Mott’s formula is marked by the dotted line.

In Fig. 4(a)we show the transmission spectra in a 4-ZNR *p* junction with two local potential ${U}_{0,1}^{1}=-\,{U}_{0,4}^{2}=t$. The spectra show symmetry set (0,1,0) and their slopes remain almost constant over a large range near the Fermi level. As a result, as shown in Fig. 4(b), ${S}_{s}$ (dashed) follows very well the Mott’s formula (dotted) with $d{S}_{s}/dT\approx 7446\mu {k}_{B}{\rm{V}}/t{\rm{K}}$ until $T\approx 0.01t/{k}_{B}$ which is around the room temperature *T* = $300{\rm{K}}$ for ZGNRs. On the other hand, ${S}_{c}$ vanishes strictly in the whole range of temperature, indicating the realization of pure thermal spin current due to the symmetry. This suggests promising application potential for thermo-spintronics in large range of temperature.

### Disorder inside nanoribbon

The electron transport in ZNRs is carried out via edge states corresponding to the energy bands near the Fermi level. Disorders inside nanoribbon have much less influence to the transmission spectra than those on edge. The transmission spectra deviate only slightly from those in pristine ZNRs if both local potentials are located inside. Nevertheless, proper disorder inside can be used to fine-tune the spectra as well as the thermoelectricity. This can be used to improving the symmetry of the transmission spectra to achieve higher thermopower performance in some cases as discussed below.

**Figure 5:**
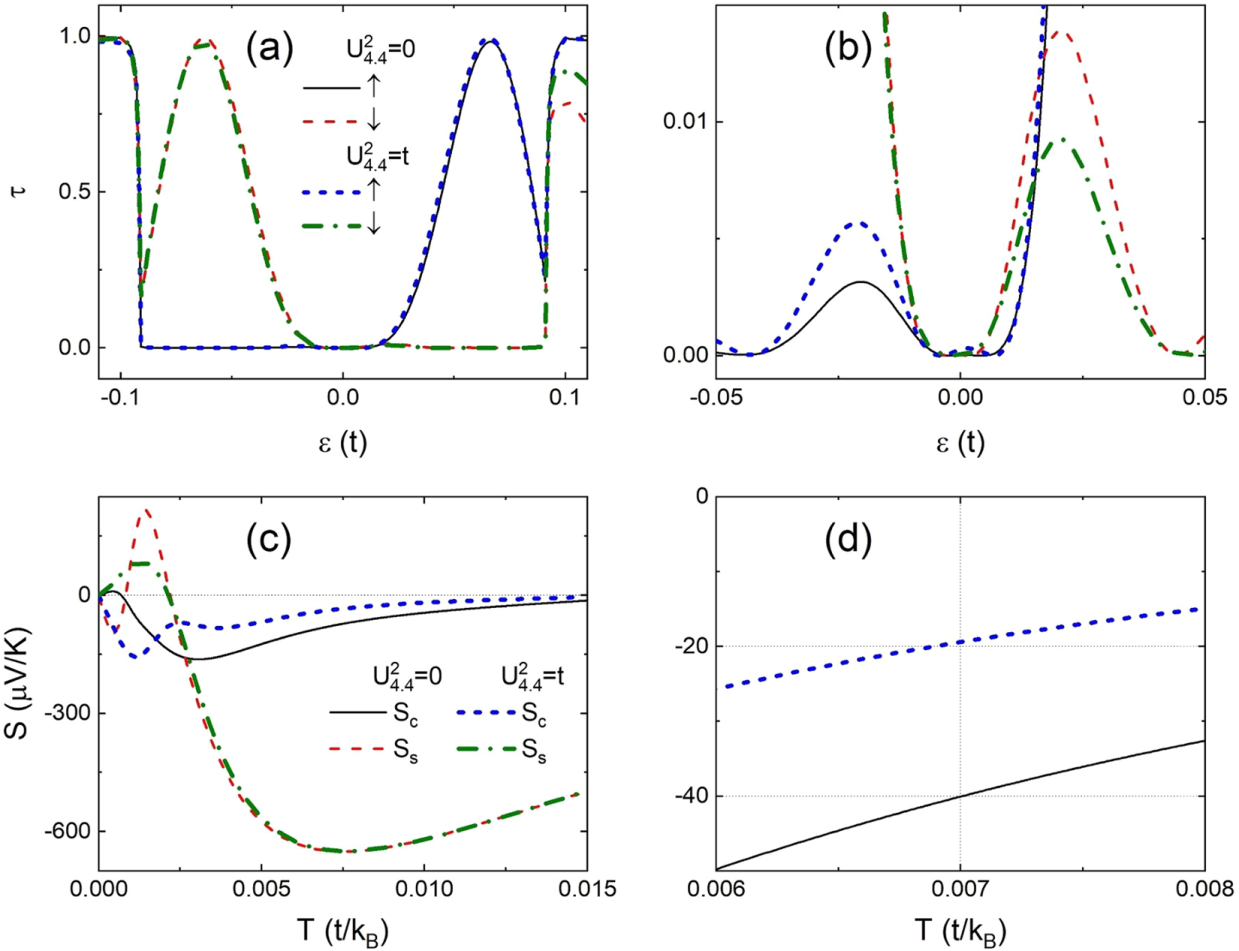
(**a**) Transmission spectra of a 10-ZNR *ap* junction for $m=5$, $M=0.1t$ and ${U}_{4,1}^{1}=t$ with (dotted and dash-dotted) and without (solid and dashed) ${U}_{4,4}^{2}$. (**b**) Zoom of (**a**) near the Fermi level. (**c**) The corresponding Seebeck coefficients versus $T$. (**d**) Zoom of (**c**) near $T=0.007t/{k}_{B}$ for the charge Seebeck coefficients.

In Fig. 5 we show the transmission spectra, (a) and (b), and Seebeck coefficients, (c) and (d), for a wider *ap* junction made of 10-ZNR for $m=5$ and $M=0.1t$. There is one local potential on edge with ${U}_{4,1}^{1}=t$ while a second is located inside with ${U}_{4,4}^{2}=t$ (dotted and dash-dotted). For the sake of comparison, we present also the results in the absence of the second potential with ${U}_{4,4}^{2}=0$ (solid and dashed).

In the absence of the second potential with ${U}_{4,4}^{2}=0$ the transmission spectra satisfy roughly symmetry ② as illustrated in Fig. 5(a). Two peaks with almost the same size appear on both sides of the Fermi level. However, as zoomed in Fig. 5(b), the curves of *τ*_↑_ and *τ*_↓_ are not strictly mirror of each other with respect to the Fermi level. The *τ*_↑_ peak at $\varepsilon =-\,0.021\,t$ (solid) is much lower than the *τ*_↓_ peak at $\varepsilon =0.021t$ (dashed). The corresponding charge Seebeck coefficient does not vanish as shown by the thin solid curve in Fig. 5(c). Though a huge ${S}_{s}$ peak is obtained around $T=0.007t/{k}_{B}$ (thin dashed), we do not have pure thermal spin current in the system.

This situation can be improved if we add the second potential inside the nanoribbon with ${U}_{4,4}^{2}=t$. It increases (reduces) the *τ*_↑_ (*τ*_↓_) peak at $\varepsilon =-\,0.021t$ ($=0.021t$) as shown by the dotted (dash-dotted) curve in Fig. 5(b). This weakens the asymmetry of the spectra and suppresses ${S}_{c}$ significantly at high temperature. At $T=0.007t/{k}_{B}$, as zoomed in Fig. 5(d), ${U}_{4,4}^{2}=t$ reduces $|{S}_{c}|$ from $40\mu {\rm{V}}/{\rm{K}}$ to $20\mu {\rm{V}}/{\rm{K}}$ while remains ${S}_{s}\approx -\,646\mu {\rm{V}}/{\rm{K}}$ intact.

### Figure of merit

**Figure 6:**
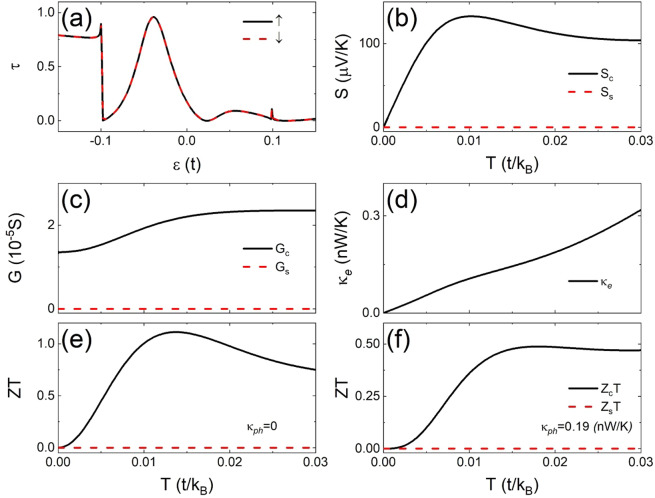
(**a**) Transmission spectra of a 4-ZNR *ap* junction with $m=5$, $M=0.1t$, and ${U}_{2,1}^{1}={U}_{-2,1}^{2}=t$ are plotted together with the temperature dependence of (**b**) Seebeck coefficient, (**c**) conductance, (**d**) electron thermal conductance, (**e**) thermoelectric figures of merit $ZT$ neglecting the phonon thermal conductance (${\kappa }_{ph}=0$), and (f) $ZT$ assuming ${\kappa }_{ph}=0.19n{\rm{W}}/{\rm{K}}$, a typical value for pristine ZGNR^6^.

In Fig. 6(a), we show the transmission spectra of a 4-ZNR *ap* junction with $m=5$, $M=0.1t$, and ${U}_{2,1}^{1}={U}_{-2,1}^{2}=t$. They are spin degenerate with symmetry set (1,0,0) and have a huge peak below the Fermi level at $\varepsilon =-\,0.4t$. A steep downhill slope passes through the Fermi level and the transmission remains relatively low at high energy. The junction shows a pure charge thermopower over $100\mu {\rm{V}}/{\rm{K}}$ at high temperature, a steady temperature dependence of conductance, and almost linear temperature of electron thermal conductance^11^ as illustrated in Fig. 6(b–d), respectively. The charge figure of merit ${Z}_{c}T$ can be larger than one near $T=0.01t/{k}_{B}$ as shown in Fig. 6(e) if the phonon thermal conductance is negligible which might be reasonable in some cases^24^. Assuming a typical phonon thermal conductance ${\kappa }_{ph}=0.19n{\rm{W}}/{\rm{K}}$ of pristine ZGNR at room temperature^6^, we obtain a steady ${Z}_{c}T\approx 0.5$ at high temperature as shown in Fig. 6(f) ${\kappa }_{ph}=0.19n{\rm{W}}/{\rm{K}}$.

**Figure 7:**
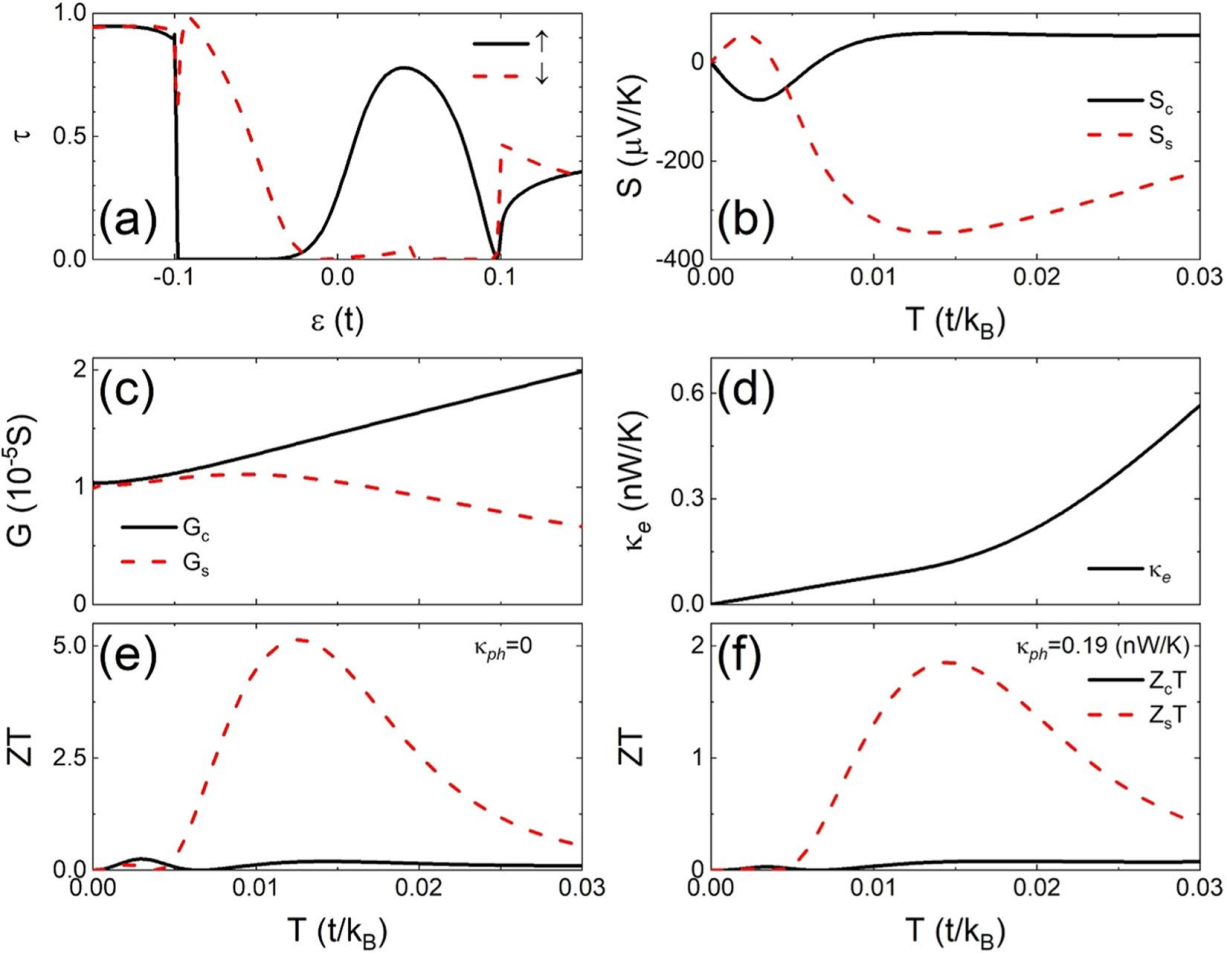
The same as Fig. (6) in disorder configuration ${U}_{5,1}^{1}={U}_{3,1}^{2}=t$.

Interestingly, using the definition of ${Z}_{s}T$ in Eq. (4), we observe that it vanishes if the transmission spectra of junction show any symmetry of ①, ②, and ③. This happens because we have ${G}_{\uparrow }={G}_{\downarrow }$ and ${G}_{s}=0$ in the presence of symmetry ① or ② while ${S}_{s}={S}_{c}=0$ for symmetry ③. Therefore, a large ${Z}_{s}T$ could be found only when symmetry ①, ②, and ③ are all broken. One case with significant ${Z}_{s}T$ value is illustrated in Fig. 7, where the transmission spectra are plotted with the temperature dependence of conductance and thermoelectric parameters in a 4-ZNR *ap* junction of $m=5$ and $M=0.1t$ with two local potential ${U}_{5,1}^{1}={U}_{3,1}^{2}=t$. At $\varepsilon =0$ both ${\tau }_{\uparrow }$ and ${\tau }_{\downarrow }$ have positive slope and ${\tau {\prime} }_{\downarrow }/{\tau }_{\downarrow }\approx 3{\tau {\prime} }_{\uparrow }/{\tau }_{\uparrow }$, so ${S}_{c}$ and ${S}_{s}$ have opposite sign but almost the same magnitude as shown in Fig. 7(a,b). In larger range of energy, however, ${\tau }_{\uparrow }$ has a wide gap below the Fermi level and a peak at $\varepsilon =0.04t$ while ${\tau }_{\downarrow }$ increases to 1 around $\varepsilon =-0.09t$ and has a wide gap above the Fermi level. This variation of ${\tau }_{\uparrow }$ and ${\tau }_{\downarrow }$ reverses the sign of ${S}_{\downarrow }$ and gives ${S}_{S}\approx -6{S}_{C}$ at $T=0.01t/{k}_{B}$. The spin conductance ${G}_{s}$ does not vanish due to the absence of symmetry ① and ② in the transmission spectra and the electronic thermal conductance ${\kappa }_{e}$ increases steadily with the temperature as illustrated in Fig. 7(c,d). As a result, a huge thermoelectric figures of merit ${Z}_{s}T > 5$ is obtained around $T=0.01t/{k}_{B}$ with relative small ${Z}_{c}T$ as shown in Fig. 7(e) if the lattice thermal conductance ${\kappa }_{ph}$ is neglected. As shown in Fig. 7(f), a peak value of ${Z}_{s}T=1.852$ at $T=0.0145t/{k}_{B}$ can be reached even if we use ${\kappa }_{ph}=0.19n{\rm{W}}/{\rm{K}}$, the value for perfect ZGNRs at room temperature^6^, which might be an overestimated value in most of the cases since ${\kappa }_{ph}$ of ZNR can be significantly reduced by physical and chemical modification^47^. Our results suggest that the GNRs with double disorder on edges have application potential for high performance spin thermoelectric devices.

### Finite temperature bias

We have seen that charge and spin thermoelectric parameters of FM ZNR junctions can be well manipulated by double local disorders in the linear regime of temperature bias. Especially locating the two disorders in the domain wall of an *ap* junction can give ample variety of the parameters and introduce desired spin polarization in the systems. In this subsection, we consider a 4-ZNR *ap* junction with the length of region C or the domain wall $m=10$ and a maximal edge Zeeman energy $M=0.15t$ when the temperature of right electrode is fixed at ${T}_{R}=0.001t/{k}_{B}$ and the strength ratio of the two local potentials reads ${U}_{7,1}^{1}=2{U}_{5,1}^{2}$. The variation of the left electrode temperature and the potential strength can also be used to control the spin polarization of current in the system.

**Figure 8:**
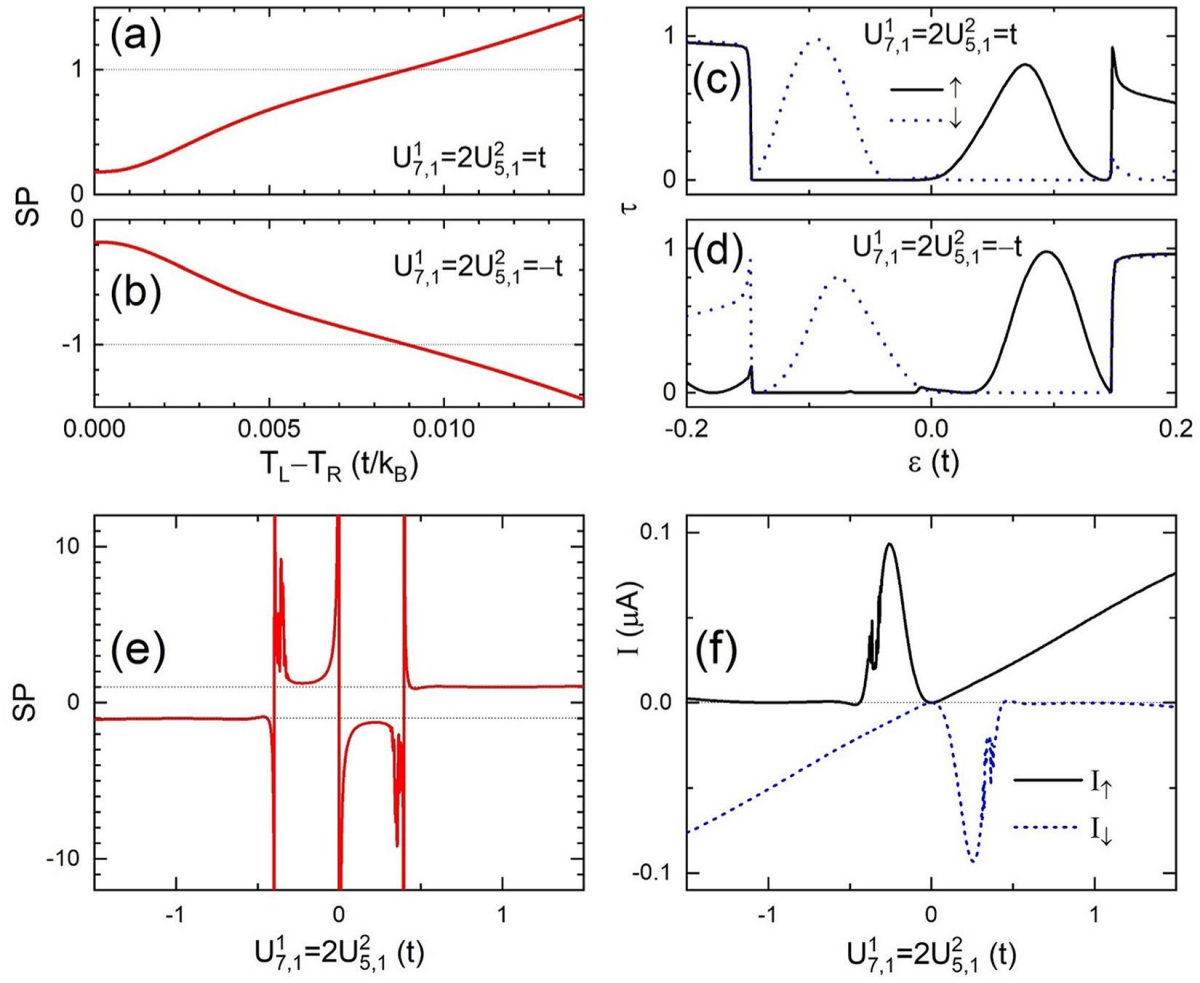
Spin polarization *SP* versus the temperature difference ${T}_{L}-{T}_{R}$ in a 4-ZNR *ap* junction with $m=10$ and $M=0.15t$ at ${T}_{R}=0.001t/{k}_{B}$ for (**a**) ${U}_{7,1}^{1}=2{U}_{5,1}^{2}=t$ and (**b**) ${U}_{7,1}^{1}=2{U}_{5,1}^{2}=-\,t$. The corresponding transmission spectra are plotted in (**c**,**d**), respectively. (**e**) Spin polarization and (**f**) the corresponding current versus the disorder strength ${U}_{7,1}^{1}=2{U}_{5,1}^{2}$ are also plotted for the same junction at ${T}_{L}=0.01t/{k}_{B}$, ${T}_{R}=0.001t/{k}_{B}$.

In Fig. 8(a) we plot $SP$ as a function of ${T}_{L}$ for the disorder strength ${U}_{7,1}^{1}=2{U}_{5,1}^{2}=t$. In the linear regime ${T}_{L}\approx {T}_{R}=0.001t/{k}_{B}$ *SP* remains almost constant at a value of 0.19. Then it turns upward around ${T}_{L}-{T}_{R}=0.001t/{k}_{B}$ and increases monotonically in the whole temperature range. At ${T}_{L}=0.01t/{k}_{B}$ $SP=1$ is achieved which means spin-down electrons do not contribute to the current. Furthermore, the spin-down electrons reverse their flowing direction leading to $SP > 1$ at ${T}_{L} > 0.01t/{k}_{B}$. When we inverse the sign of the disorder or set the local potential ${U}_{7,1}^{1}=2{U}_{5,1}^{2}=-\,t$, *SP* changes its sign and the *SP* curve turns upside down as shown in Fig. 8(b). Note that the sign inversion of disorder strength can be realized by change the doping type between *n* and *p* in case of doping disorder^20^. So we can control the spin type of the thermoelectric system by choosing the doping type. This happens because the spin up (down) current is mainly carried by electrons (holes) in a large range of energy as illustrated by the transmission spectra in Fig. 8(c,d) for potential strength ${U}_{7,1}^{1}=t$ and ${U}_{7,1}^{1}=-\,t$, respectively. ${\tau }_{\uparrow }$ (${\tau }_{\downarrow }$) has a big peak above (below) the Fermi level but vanishes below (above) in the energy range between $-0.15t < \varepsilon  < 0.15t$. In addition we observe another symmetry of the spectra with ${\tau }_{\sigma }(\varepsilon )$ at ${U}_{7,1}^{1}=t$ being equal to ${\tau }_{\bar{\sigma }}(\,-\,\varepsilon )$ at ${U}_{7,1}^{1}=-\,t$. This spectra result in the sign inversion of $SP$ with the sign inversion of disorder potential $U$.

A full picture of the $SP$ dependence on the disorder strength is shown in Fig. 8(e), where $SP$ versus ${U}_{7,1}^{1}$ at ${T}_{L}=0.01t/{k}_{B}$ is plotted and we have approximately $SP({U}_{7,1}^{1})=-\,SP(-{U}_{7,1}^{1})$. Pure spin current with infinite $SP$ might be achieved near ${U}_{7,1}^{1}=-\,0.4t$, $0$, and $0.4t$. This corresponds to opposite flow directions of spin-up and spin-down electrons so ${I}_{\uparrow }+{I}_{\downarrow }=0$ as shown in Fig. 8(f). In cases of high disorder strength $|{U}_{7,1}^{1}| > 0.4t$, one of the spin channel is blocked and we always have the same type of spin current with ${I}_{S}={I}_{\uparrow }-{I}_{\downarrow } > 0$. For negative $U$ we have negative charge current with ${I}_{C}={I}_{\downarrow } < 0$ and $SP\approx -\,1$ while for positive $U$ we have positive charge current with ${I}_{C}={I}_{\uparrow } > 0$ and $SP\approx 1$. The above properties allow us to design and control spin polarized current in a convenient and highly responsive way, which has great value on the application in spintronics.

## Conclusion

Employing the nonequilibrium Green functions in the tight-binding model, we have studied the thermoelectric properties of graphene-like zigzag nanoribbons modified by two on-site disorder potentials. We emphasize that the characteristic thermopower in a two-probe junction is closely relevant to the symmetry of its transmission spectra, and also to the geometry configuration of the host material and the disorders. Junctions of even-width FM ZNRs with antiparallel electrode magnetizations have shown ample variation for subtle manipulation. Choosing properly the locations of two edge disorders, we can obtain spin-up and spin-down transmission spectra with desired symmetries such as ① spin symmetry, ② mutual mirror symmetry, and ③ mirror symmetry. Pure charge thermal current appears in case of transmission spectra with only symmetry ①. Pure spin thermal current appears in case of transmission spectra with only symmetry ②. No current can be observed in case of transmission spectra with symmetry ③ or both symmetry ① and ②. However, to obtain high ${Z}_{s}T$ value, we need to break slightly symmetry ② and get a finite spin conductance. Assuming a lattice thermal conductance ${\kappa }_{ph}=0.19n{\rm{W}}/{\rm{K}}$ estimated from perfect ZGNR, we obtain ${Z}_{c}T\approx 0.5$ and ${Z}_{s}T\approx 2$ in some ZNRs modified by double edge disorders. Optimistically, ${Z}_{c}T > 1$ and ${Z}_{s}T > 5$ might be available if the lattice thermal conductance can be suppressed by the disorders or other modifications. Disorder potentials of opposite sign work on different types of carriers similar to *n-* and *p-*type dopings in semiconductors. This suggests another possible symmetry in the system and might offers an extra degree of freedom for the spin polarization manipulation of thermal current. Different from edge modification, disorders inside ZNRs can have limited effects on thermopower and can be used to finely tune the thermoelectric properties.
